# Supplementary material for: RNAi Screen Identifies AXL Inhibition Combined with Cannabinoid WIN55212-2 as a Potential Strategy for Cancer Treatment
Source: Pharmaceuticals (Basel). 2024 Nov 1;17(11):1465. doi: 10.3390/ph17111465 (PMC11597789; doi:10.3390/ph17111465)
Supplement: Supplementary file 1 [file pharmaceuticals-17-01465-s001.zip › pharmaceuticals-3232234-supplementary.pdf]

**Table S1.** The 49 of targeted genes that synergistically inhibit cancer cell viability with cannabinoid WIN55, 212-2 using siRNA screening.

| Genes | Proliferation rate SiRNA group | Proliferation rate SiRNA plus WIN group | Synergistic activity (SA) |
|---|---|---|---|
| AKAP8 | 0.942 | 0.711 | 0.231 |
| SNX16 | 0.953 | 0.79 | 0.163 |
| RNASEL | 0.950 | 0.805 | 0.145 |
| TAOK2 | 0.959 | 0.821 | 0.138 |
| STK4 | 0.935 | 0.802 | 0.134 |
| LRRK2 | 0.977 | 0.851 | 0.126 |
| PRKCD | 0.976 | 0.861 | 0.115 |
| SKP2 | 0.998 | 0.891 | 0.107 |
| CKB | 0.992 | 0.887 | 0.105 |
| RPS6KA3 | 0.983 | 0.882 | 0.101 |
| TNK1 | 0.727 | 0.629 | 0.098 |
| CDC2L5 | 0.923 | 0.827 | 0.096 |
| MKNK1 | 0.950 | 0.857 | 0.093 |
| PDGFRB | 0.99 | 0.898 | 0.091 |
| FN3K | 0.929 | 0.838 | 0.091 |
| RIPK2 | 1.053 | 0.800 | 0.253 |
| ITK | 1.053 | 0.865 | 0.188 |
| CDKL5 | 1.023 | 0.834 | 0.188 |
| IHPK3 | 1.047 | 0.859 | 0.187 |
| SRMS | 1.001 | 0.836 | 0.165 |
| PLK3 | 1.078 | 0.916 | 0.162 |
| MAPK8 | 1.022 | 0.860 | 0.162 |
| PRKCI | 1.031 | 0.876 | 0.155 |
| AXL | 1.027 | 0.877 | 0.151 |
| MAPK7 | 1.01 | 0.861 | 0.149 |
| PCTK2 | 1.01 | 0.862 | 0.148 |
| PDK2 | 1.074 | 0.93 | 0.145 |
| CLK2 | 1.086 | 0.944 | 0.142 |
| CDK10 | 1.038 | 0.896 | 0.142 |
| MAPK6 | 1.024 | 0.886 | 0.138 |
| CSNK2A2 | 1.042 | 0.907 | 0.134 |
| CIB4 | 1.000 | 0.87 | 0.13 |
| STK17B | 1.002 | 0.873 | 0.128 |
| SH3BP5 | 1.053 | 0.926 | 0.127 |
| ACVR2B | 1.043 | 0.915 | 0.127 |
| PCTK3 | 1.047 | 0.928 | 0.12 |
| PKLR | 1.057 | 0.938 | 0.119 |
| BUB1B | 1.013 | 0.907 | 0.107 |
| ATR | 1.012 | 0.907 | 0.105 |

| | | | |
|---|---|---|---|
| PDGFRL | 1.052 | 0.950 | 0.102 |
| CLK3 | 1.053 | 0.953 | 0.1 |
| SH3BP4 | 1.008 | 0.908 | 0.1 |
| PRKCSH | 1.063 | 0.964 | 0.099 |
| ERN1 | 1.006 | 0.909 | 0.097 |
| MARK3 | 1.047 | 0.952 | 0.096 |
| ACVR1B | 1.015 | 0.923 | 0.092 |
| GRK6 | 1.047 | 0.956 | 0.091 |
| CALM3 | 1.011 | 0.921 | 0.091 |
| MINK1 | 0.892 | 0.800 | 0.092 |

Synergistic activity (SA), was calculated with formula as follow: SA= Inhibition Rate (siRNA)-Inhibition Rate (siRNA + WIN 55,212-2). The potential synergistic genes involved into the cancer pathway were marked in red.

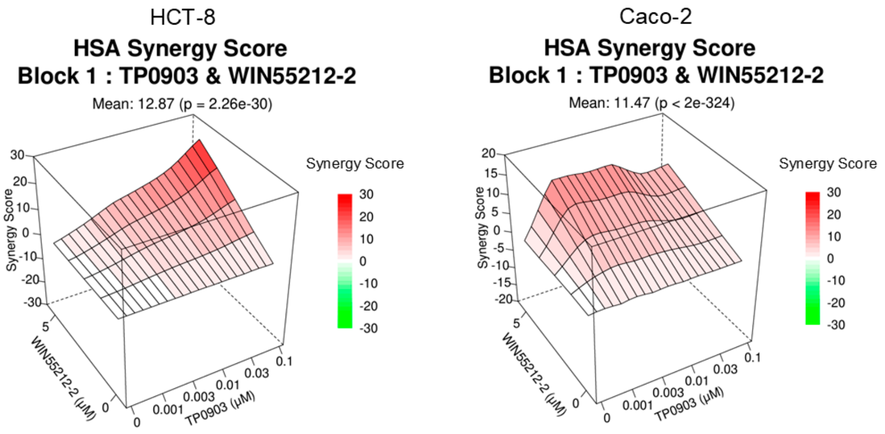


**Figure S1.** The Synergy Score of TP0903 plus WIN55212-2 treatment in HCT-8 and Caco-2 cells using the HSA model in SynergyFinder 3.0 website analysis.

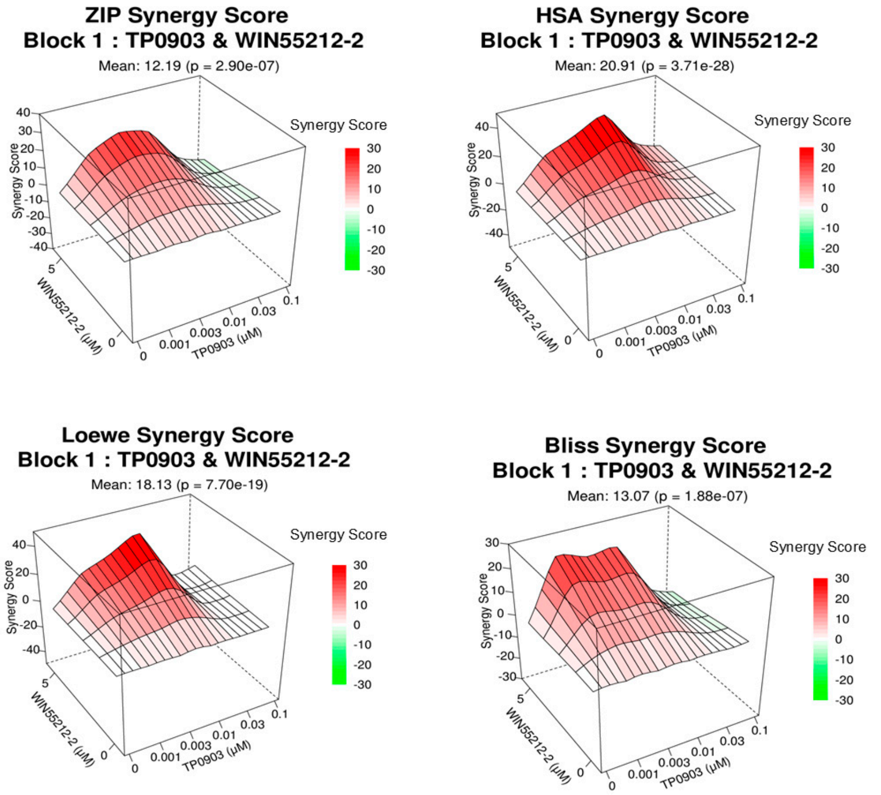


**Figure S2.** The Synergy Score of TP0903 plus WIN55212-2 treatment in HCT-8 cells using the ZIP, HAS, Loewe and Bliss models in SynergyFinder 3.0 website analysis.

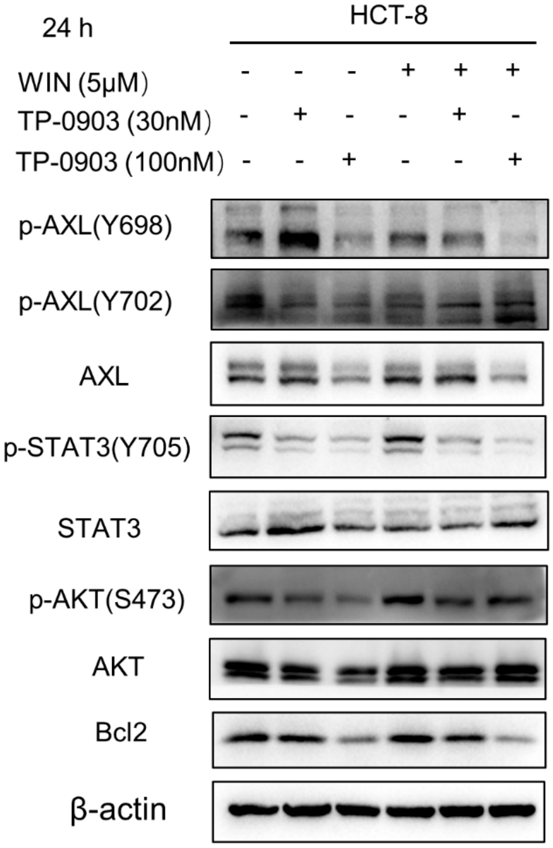


**Figure S3.** HCT-8 cells were treated with TP-0903 (30 nM or 100 nM) alone or in combination with 5 μM of WIN55212-2 for 24 h. The expressions of phosphorylated AXL (p-$AXL_{Tyr698}$, p-$AXL_{Tyr702}$), phosphorylated AKT (p-$AKT_{Ser473}$), phosphorylated STAT3 (p-$STAT3_{Tyr705}$), AXL, AKT, STAT3 and BCL2 were evaluated by western blot analysis.

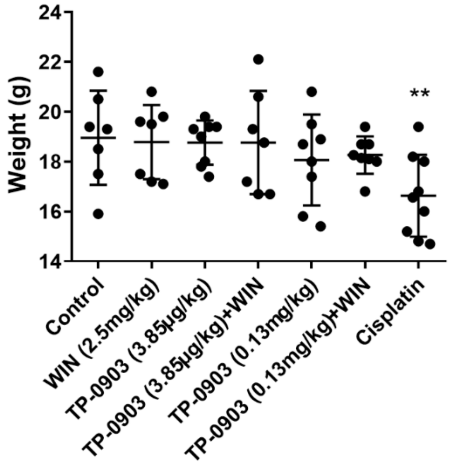


**Figure S4.** BALB/c nude mice were injected subcutaneously in the flank with HCT-8 colon cancer pieces and intratumorally treated with TP-0903 (3.85 μg/kg or 0.13 mg/kg) alone or in the presence of WIN55212-2 (2.5 mg/kg) every two days. Cisplatin was used as control group (5 mg/kg per week). The body weights of individual mice were detected.
